# MicroRNA-185-5p targets tyrosine 3-monooxygenase/tryptophan 5-monooxygenase activation protein zeta to regulate non-small cell lung cancer progression

**DOI:** 10.1186/s13019-023-02342-x

**Published:** 2023-07-31

**Authors:** Jiangang Ma, Yan Bai, Fangyuan Chen, Feng Zhou, Liyuan Zhang, Peini Xue, Dong Wang

**Affiliations:** Department of Respiratory and Critical Care Medicine, The Second Affiliated Hospital of Shaanxi University of Chinese Medicine, No.5 Weiyang West Road, Qindu District, Xianyang, 712000 Shaanxi China

**Keywords:** NSCLC, miRNAs, miR-185-5p, YWHAZ, Molecular mechanism

## Abstract

**Background:**

Lung cancer (LC) is one of the most frequent cancers worldwide, as well as the leading cause of cancer-related death. Non-small cell lung cancer (NSCLC, which accounts for 85% of occurrences) is the main type of LC. MiRNAs appear to play a role in the occurrence and progression of many malignancies, according to mounting data. The underlying mechanism of miRNAs in regulating NSCLC cell biological activity and progression, on the other hand, is still being investigated.

**Methods:**

QRT-PCR were used to detect miR-185-5p expression and YWHAZ mRNA in NSCLC. The CCK-8 assay was used to determine the tumor cells’ ability to proliferate. Transwall assay was used to test the migratory and invasive properties of cells. Cell apoptosis was detected using flow cytometry. Tyrosine 3-monooxygenase/tryptophan 5-monooxygenase activation protein zeta (YWHAZ), E-Cadherin, N-Cadherin and cleaved-caspase3 protein expression were assessed using Western Blot. The bioinformatics analysis software StarBase2.0 predicted miR-185-5p downstream targets. To confirm the target association between miR-185-5p and YWHAZ, a luciferase experiment was used. In addition, an NCl-H1299 xenograft model was created to assess the anti-tumor impact of miR-185-5p in vivo. The expression level of YWHAZ in tumor tissues of small xenograft tumor model was detected by immunohistochemistry assay.

**Results:**

Decreased miR-185-5p expression levels were observed in NSCLC. In vitro, over-expressed miR-185-5p decreased cell viability, proliferation, invasion/migration, and induced cell apoptosis, while inhibiting tumor growth in vivo. Dual-luciferase gene experiments confirmed that YWHAZ binds to miR-185-5p. Overexpression of YWHAZ partially restored the inhibitory effects of miR-185-5p on cell behaviors.

**Conclusion:**

MiR-185-5p was down-regulated in NSCLC, and that overexpressed miR-185-5p inhibited malignant behaviors of cells and tumor growth by negatively regulating YWHAZ.

## Introduction

Lung cancer (LC) is one of the most frequent cancers worldwide, as well as the leading cause of cancer-related death. According to global cancer statistics, more than 1.6 million people died of lung cancer around the world in 2018, and the number of deaths has been rising in recent years [1]. Non-small cell lung cancer (NSCLC, which accounts for 85% of occurrences) and small cell lung cancer (SCLC, which accounts for 15% of cases) are the two main types of lung cancer [1]. Because of its late discovery and low susceptibility to chemotherapy and radiotherapy, NSCLC, the most common kind of lung cancer, is a particularly difficult malignancy to treat [2]. Currently, the 5-year overall survival of NSCLC patients is still as low as 15% [3], 50–60% of NSCLC patients develop metastases during treatment, and up to 30% of patients develop metastases, recurrences and metastases at diagnosis is a major factor in poor prognosis [4]. As a result, identifying new therapeutic targets and characterizing the potential molecular mechanisms of NSCLC is critical in order to improve the low survival rate of NSCLC patients.

MicroRNAs (MiRNAs) are a type of endogenous noncoding RNA with regulatory activities of gene expression found in eukaryotes. They have a length of 18–25 nucleotides (nt) [5]. MiRNAs have been identified to influence the development and progression of different malignancies, including NSCLC, by functioning as tumor initiators or suppressors. For example, down-regulated miR-22-3p targets PGC1β and promotes tumorigenesis of cells through the PPARγ signaling pathway in breast cancer [6]. MiR-18a-5p is upregulated in hepatocellular carcinoma, and its overexpression significantly promotes tumor cell growth, while miR-18a-5p acts as an oncogenic factor to accelerate the malignant phenotype of hepatocellular carcinoma by inhibiting CPEB3 [7]. Notably, several studies in recent years have found that miR-185-5p is deregulated in a range of malignancies, including breast, prostate, and lung cancers [8–10]. MiR-185-5p, for example, can regulate cell apoptosis in breast cancer by targeting BCL2, and could be used as a therapeutic option in the treatment of breast cancer [11]. The expression of miR-185-5p, an anti-metastatic miRNA, is down-regulated in liver cancer, and up-regulating the expression of miR-185-5p can block the migration and invasion capacity of liver cancer cells [12]. Recent evidence suggests that miR-185-5p affects NSCLC by targeting the RAB35 gene, which is mediated by cell-derived exosomes [13]. However, the fundamental mechanism of miR-185-5p in affecting NSCLC cell biological behavior and progression remains to be further studied.

In this study, bioinformatics analysis suggested that tyrosine 3-monooxygenase/tryptophan 5-monooxygenase activation protein zeta (YWHAZ) might be an underlying target of miR-185-5p. We speculated that miR-185-5p plays a tumor-suppressive role in NSCLC and miR-185-5p/YWHAZ axis was involved in NSCLC progression. The aim of this study is to explore the expression, functions and potential mechanisms of miR-185-5p in NSCLC. Eventually, as experiments suggested, downregulated miR-185-5p inhibited malignant progression of NSCLC by negatively regulating YWHAZ.

## Material and methods

### Patients and tissue samples collection

The Second Affiliated Hospital of Shaanxi University of Chinese Medicine Hospital's Institutional Research Ethics Committee (S2020-JC-YB-0123) has given us permission to conduct the study. At the Shaanxi University of Chinese Medicine Hospital's Second Affiliated Hospital, 38 pairs of fresh NSCLC tissues and adjacent normal tissues were collected from patients after they were diagnosed by experimented pathologists and gave their consent to have their tissue samples used for scientific research (Shanxi, China). The resected tissue samples were promptly frozen and kept at a temperature of 80 °C until they were used. Furthermore, the ethics committee also gave the approval for animal experiments.

### Cell culture

The National Collection of Authenticated Cell Cultures (Shanghai, China) provided human NSCLC cell lines (NCl-H322, A549, NCl-H1299, and PC9 cells) as well as human bronchial epithelial cell lines (BEAS-2B). All cell lines were grown in RPMI-1640 medium (Sigma-Aldrich, USA) with 10% fetal bovine serum (Gibco, USA) and 1% (w/v) penicillin/streptomycin (Sigma-Aldrich, USA). Cell cultures were kept in a 5% CO_2_ cell incubator with a humidified atmosphere at 37 °C.

### RNA isolation

Total RNA was extracted and purified from frozen tissues and cells using the TRIzol kit (Invitrogen, USA). UV–VIS Spectrophotometry was used to determine the concentration and quality of RNA using a Nanodrop ND-1000 (Thermo Fisher Scientific).

### Quantitative reverse transcription-PCR (qRT-PCR)

After extracting total RNA, the level of RNA transcripts was detected by the qRT-PCR analysis. Briefly, the Transcriptional First Strand cDNA Synthesis Kit (Applied Biosystems, USA) was used to reverse transcribe miR-185-5p into cDNA. Following that, SYBR Green Master Mix (Bio-Rad, USA) was used to conduct qRT-PCR. The 2^−ΔΔCq^ method was used to standardize the relative expression of miRNA and mRNA in each sample. As controls, the U6 or GAPDH was applied, respectively. The primers that were generated (Genscript Nanjing Inc., China) as follows:

miR-185-5p: Forward: 5′-CGCTGGAGAGAAAGGCAGT-3′;
  Reverse: 5′-GTGCAGGGTCCGAGGT-3′.
YWHAZ: Forward: 5′-TGTAGGAGCCCGTAGGTCATC-3′;
  Reverse: 5′-GTGAAGCATTGGGGATCAAGA-3′.
U6: Forward: 5′-CTCGCTTCGGCAGCACA-3′;
  Reverse: 5′-AACGCTTCACGAATTTGCGT-3′.
GAPDH: Forward: 5′-AGATCATCAGCAATGCCTCCT-3′;
  Reverse: 5′-TGAGTCCTTCCACGATACCAA-3'.

### Cell transfection

RiboBio Co., Ltd (Guangzhou, China) designed and supplied the miR-185-5p mimic, miR-185-5p inhibitor and its negative control (miR-NC). YWHAZ overexpression vector (pcDNA-YWHAZ) and its corresponding negative control (pcDNA) were cloned into pcDNA by RiboBio Co., Ltd. Then, we planted cells into 12-well plates and transfected cells with miR-185-5p mimic, miR-185-5p inhibitor, pcDNA-YWHAZ or their corresponding negative controls after 24 h incubation by Invitrogen™ Lipofectamine 3000 (Life Technologies, USA).

### Cell proliferation assay

A Cell Counting Kit-8 (CCK-8) test was used to determine the ability of cells to proliferate. We collected cells, which has been transfected with miR-185-5p, pcDNA-YWHAZ or their corresponding negative controls, and planted 3 × 10^4^ cells into each well of 96-well plates and then cultured at 37 °C in a 5% CO_2_ cell incubator for 24, 48 or 72 h. After that, we added 10 µL CCK-8 solutions (Sigma, USA) into each well and incubated the plates at 37 °C for another 2 h. The absorbance in each well of the plates were measured using a microplate reader at 450 nm.

### Cell migration and invasion assay

A Transwell chamber (8 m pore size; Corning, USA) coated with Matrigel matrix was used to measure cell invasion. Firstly, we collected the transfected cells and prepared a 1 × 10^5^ cells/mL cell suspension by resuspending cells with serum-free RPMI-1640. Subsequently, 200 µL cell suspension and 600 µL RPMI-1640 medium supplemented with 10% FBS were added to the upper and lower chamber respectively. After incubation for 48 h in a 5% CO_2_ cell incubator at 37 °C, we fixed the invaded cells with methanol and then stained them using 1% crystal violet. At last, the invaded cells were counted with a microscope (Olympus, Japan). The cell migration assay followed the same protocol as the cell invasion assay, with the exception that the transwell chambers used for the cell migration assay without covering with Matrigel matrix (8 μm pore size; Corning, USA).

### Cell apoptosis assay

The Annexin V-FITC/PI kit (BD Biosciences, USA) was used to measure cell apoptosis. Briefly, transfected NCl-H1299 and PC9 cells after 72 h incubation, cells were harvested with the use of 0.25% trypsinization, and then centrifuged at 3000 rpm for 15 min to collect cells. After washing with pre-chilled 1× PBS, cells were prepared into the cell suspension with 500 μL 1× binding buffer. The cells were then incubated in the dark for another 15 min at 4 °C with 5 μL Annexin V-FITC and 5 μL PI added to the cell suspension. Subsequently, flow cytometry (BD Biosciences, USA) was used to analyze the samples.

### Target prediction and dual-luciferase reporter gene assay

StarBase2.0 was used to predict the potential target genes of miR-185-5p. We purchased wild-type (WT) and mutant (MUT) YWHAZ plasmids from RiboBio Co., Ltd (Guangzhou, China). Briefly, we cloned the predicted 3′UTR sequences of WT-YWHAZ and MUT-YWHAZ both containing a binding site for miR-185-5p into the pGL3 vector respectively, to construct YWHAZ-WT and YWHAZ-MUT vector. For the dual-luciferase reporter assay, we planted 1 × 10^5^ cells into each well of 12-well plates, and then used Lipofectamine 3000 reagent to co-transfect these recombinant vectors and miR-185-5p or miR-NC into NCl-H1299 and PC9 cells according to the manufacturers’ instruction. After 48 h transfection, we collected cells, and then lysed cells with 1 × PLB lysis buffer (Sigma-Aldrich, USA). A dual luciferase reporter gene assay system (BD Biosciences, USA) was used to assess luciferase activity.

### Western blot

Western blot was used to detect YWHAZ, E-Cadherin, N-Cadherin and cleaved-caspase3 expression. Briefly, we harvested transfected NCl-H1299 and PC9 cells with 0.25% trypsinization, and centrifuged for 15 min at 3000 rpm. Total proteins in cells were extracted by RIPA lysis buffer (Beyotime, China) and then measured using a Nano-Drop 2000c spectrophotometer (Thermo Fisher Scientific, USA). After separated by 10% SDS-PAGE gel electrophores in equal amounts, proteins were transferred onto polyvinylidene difluoride (PVDF) membranes. Then, we blocked the PVDF membranes using 5% skim milk at room temperature for 60 min and probed the PVDF membranes by primary antibody for a night at 4 °C. After that, the PVDF membranes was washed with PBS and co-incubated with the corresponding secondary antibodies at room temperature for 1 h. Specific protein signals were detected after washing with PBS for three times using ECL Chemiluminescent Substrate Reagent Kit (Thermo Fisher Scientific, USA). Imaging system (Bio-Rad, USA) was performed to analyze protein bands. To normalize relative protein expression levels, GAPDH expression was employed as an internal reference.

### Xenograft tumor models

The Animal Center (Nanjing Medical University, Nanjing, China) provided SPF BALB/c male nude mice (4–6 weeks old) that were raised in an SPF environment. Xenograft tumor models were constructed by subcutaneously injecting 200 μL cell suspension into the left flank of mice, which was prepared by suspending stably transfected NCl-H1299 cells in PBS (with the density of 5 × 10^6^/mL). Digital calipers were used to measure the length and width of tumors every 5 days. Tumor volumes were calculated using the following formula: 0.5 × length × width^2^ (mm^3^). The xenograft mice were euthanized after 25 days, and the tumors were excised and photographed. For the qRT-PCR, immunohistochemistry and western blot experiment, a portion of the tumor was separated and kept at 80 °C.

### Immunohistochemistry staining

The expression of YWHAZ in xenograft tumors was examined by IHC staining. Briefly, tumor tissues were embedded in paraffin after being treated with 4% paraformaldehyde. After being cut into 4 µm sections, tumor tissues were made into tissue sections, and then were deparaffinized, dehydrated, and rehydrated. Subsequently, tissue sections were incubated in 0.01 M citrate buffer at 95 °C for 20 min for antigen retrieval and took out for washing once with distilled water and twice with TBS after cooling at room temperature. To suppress endogenous peroxidase activity, 3% hydrogen peroxide was added dropwise to each tissue section and incubated for 15 min at a room temperature. After incubation, 3% hydrogen peroxide was removed. Blocked with 5% goat serum for 30 min at room temperature, tissue sections were incubated with an antibody against YWHAZ (ABGENT, USA) overnight at 4 °C and incubated with goat anti-rabbit IgG (Abcam, USA) for 30 min at room temperature, and re-stained with the chromogenic reagent 3,3′-diaminobenzidine (DAB) and hematoxylin (Sigma-Aldrich, USA). Finally, the protein expression of YWHAZ in tumor tissues was observed under a microscope (Nikon Microsystems, China) at an appropriate magnification (100×).

### Statistical analysis

SPSS 20.0 was used to analyze all of the data. The data was presented in the form of means ± standard deviation (SD). Student’s t test was used to determine statistical significance. Statistical significance is defined as *p* < 0.05.

## Results

In this study, we detected the expression level of miR-185-5p in NSCLC tissues and cells for verifying the characteristic expression in NSCLC. Next, we performed a series of experiments to investigate the regulatory function of miR-185-5p in NSCLC in vivo and in vitro, and explore the regulatory mechanism of miR-185-5p/YWHAZ axis, which were aimed to help clarify the mechanism of NSCLC progression.

### MiR-185-5p is downregulated in NSCLC

**Fig. 1:**
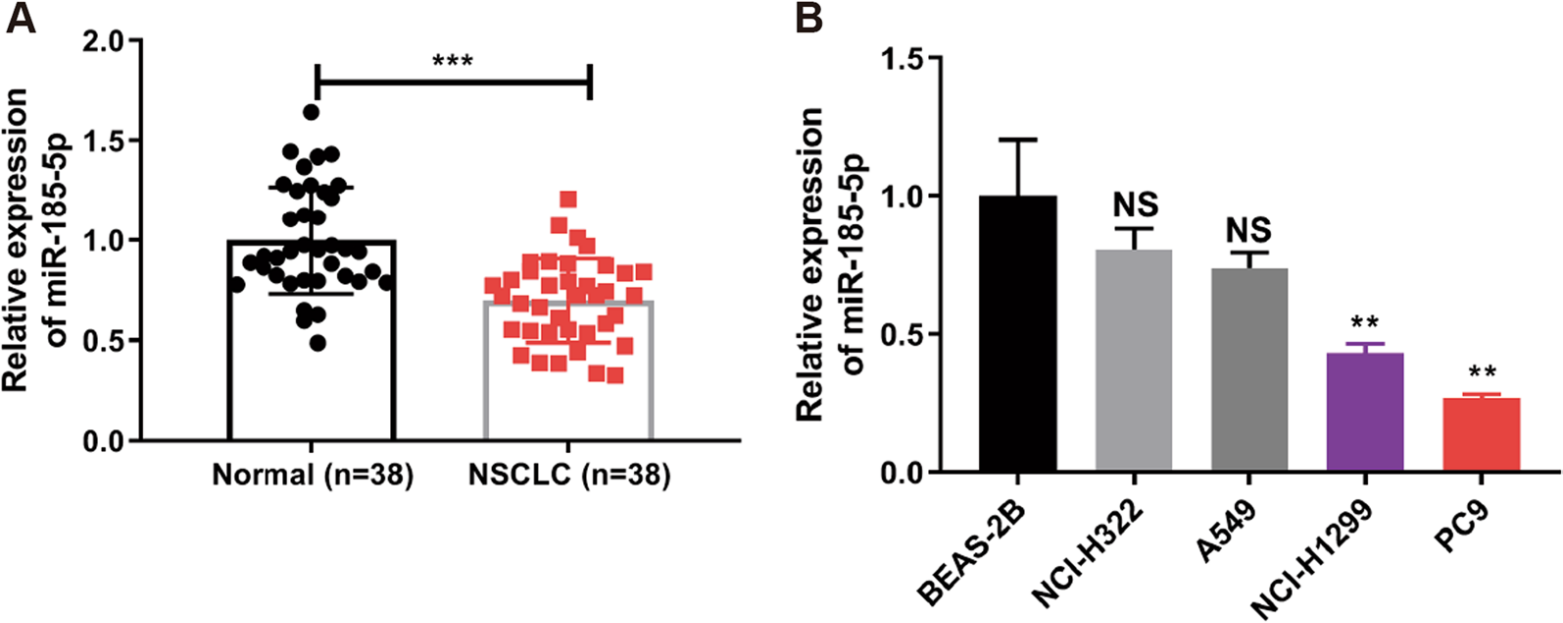
miR-185-5p is downregulated in NSCLC. **A** miR-185-5p expression in NSCLC tissues. **B** miR-185-5p expression in NSCLC cells (NCl-H322, A549, NCl-H1299 and PC9) and normal bronchial epithelium cells (BEAS-2B). Each experiments included three biological replicates. The data was displayed as Mean ± SD. **p < 0.01, ***p < 0.001

To investigate the potential dysregulation of miR-185-5p in NSCLC, RT-qPCR was used to measure the expression of miR-185-5p. Compared to nearby normal tissues, the expression of miR-185-5p was considerably down-regulated in NSCLC tissues (*p* < 0.001) (Fig. 1A). Next, we measured the expression of miR-185-5p in normal bronchial epithelium cell (BEAS-2B) and NSCLC cell lines (NCl-H322, A549, NCl-H1299 and PC9). In compared to BEAS-2B cells, miR-185-5p expression was down-regulated in NSCLC cells, particularly in NCl-H1299 and PC9 cells (*p* < 0.01) (Fig. 1B). Therefore, we chose NCl-H1299 and PC9 cells for further studies. These findings revealed that miR-185-5p expression was significantly reduced in NSCLC.

### Upregulation of miR-185-5p suppresses the cell proliferation, migration, invasion and promotes cell apoptosis of NSCLC cells

**Fig. 2:**
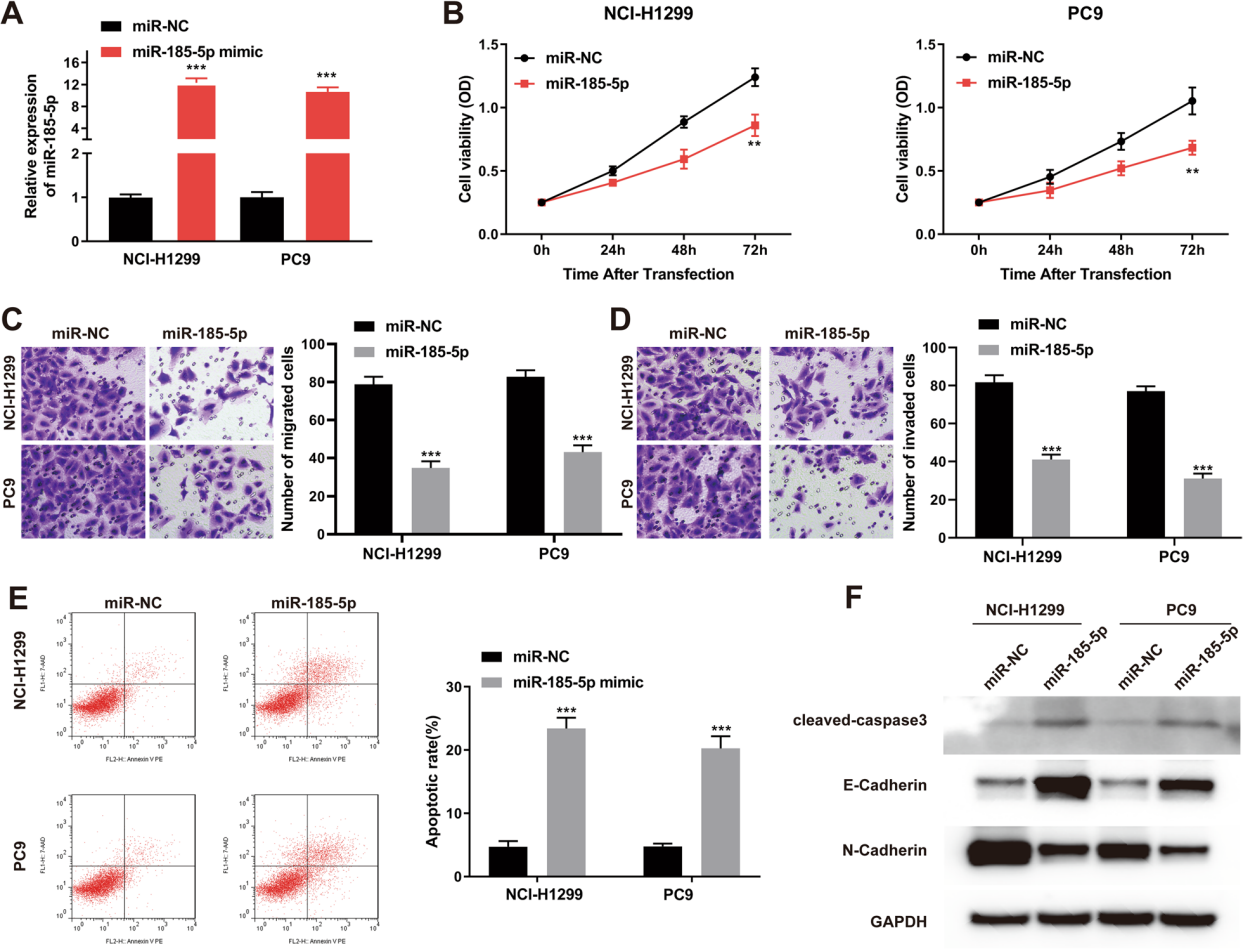
miR-185-5p overexpression inhibits cell proliferation, migration, invasion and promoted cell apoptosis of NSCLC cells. **A** miR-185-5p expression level in NCI-H1299 and PC9 after miR-185-5p mimic transfection was measured by qRT-PCR. **B** CCK-8 assay was used to assess the proliferation ability of NCI-H1299 and PC9 after miR-185-5p overexpression. **C** and **D** Transwell assay was used to assess the migration (**C**) and invasion (**D**) ability of NCI-H1299 and PC9 after miR-185-5p overexpression. **E** Cell apoptosis was measured by flow cytometry in NCI-H1299 and PC9 after miR-185-5p overexpression. **F** E-Cadherin, N-Cadherin and cleaved-caspase3 expression were measured by western blot in NCI-H1299 and PC9 after miR-185-5p overexpression. Each experiments included three biological replicates. The data was displayed as Mean ± SD. **p < 0.01, ***p < 0.001

To explore the biological function of miR-185-5p in NSCLC, we up-regulated the miR-185-5p expression level by transfecting miR-185-5p mimic into NCl-H1299 and PC9 cells. RT-qPCR analysis was used to confirm the effectiveness of miR-185-5p up-regulation (Fig. 2A). Forty-eight hours after transfection, cells were collected for performing assays. The CCK-8 assay was used to detect the effect of miR-185-5p on NSCLC cell growth. Overexpression of miR-185-5p obviously suppressed cell proliferation capacity of both NCl-H1299 and PC9 cells, as compared with the cells transfecting with the miR-NC (*p* < 0.01) (Fig. 2B). The number of invasive and migratory cells in the miR-185-5p group was much lower than in the NC group, according to the transwell assay (Fig. 2C, D). Furthermore, as E-cadherin and N-cadherin are two transmembrane glycoprotein that link cancer cells with invasion and metastasis. The western blot assay was adopted to verify the expression of E-cadherin and N-cadherin after miR-185-5p was overexpressed in NSCLC cells. Results showed that after miR-185-5p was up-regulated, the expression of E-cadherin was increased while the expression of N-cadherin was decreased (Fig. 2F). The results above showed that up-regulation of miR-185-5p significantly suppressed the invasion and migration of NSCLC cells. The flow cytometry assay was used to investigate the effect of miR-185-5p overexpression on NSCLC cell apoptosis. MiR-185-5p overexpression raised the relative cell apoptosis percentage in both NCl-H1299 and PC9 cells, as seen in Fig. 2E. In addition, western blot analysis also indicated that overexpression of miR-185-5p induced the up-regulation of cleaved-Caspase3 (Fig. 2F). Thus, these results demonstrated that upregulation of miR-185-5p suppressed cell proliferation, migration, invasion and promoted cell apoptosis of NSCLC cells.

### YWHAZ is a direct downstream target of miR-185-5p

**Fig. 3:**
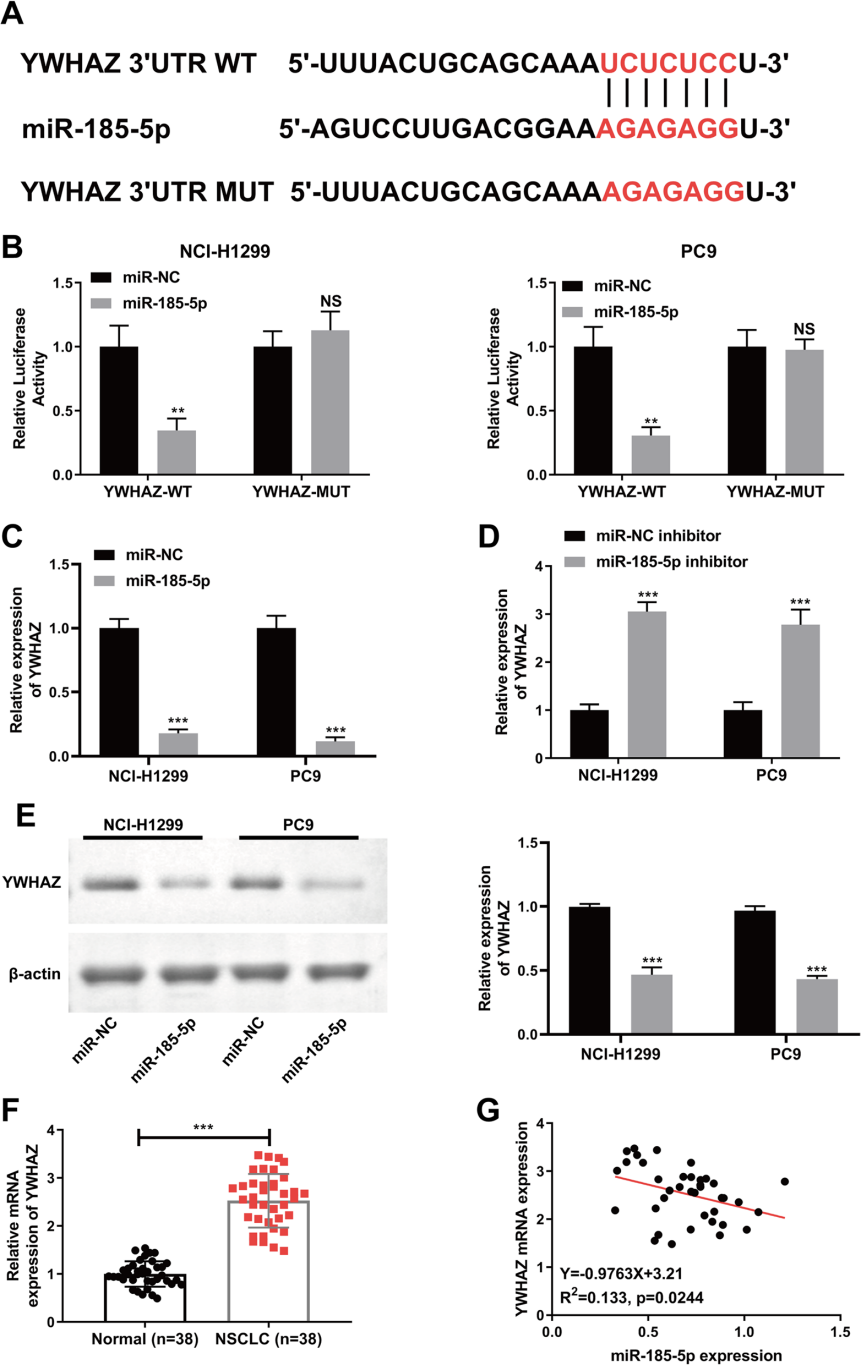
YWHAZ is a direct downstream target of miR-185-5p in NCI-H1299 and PC9 cells. **A** StarBase2.0 was used to predict the potential binding sequences between YWHAZ and miR-185-5p. **B** Dual-luciferase reporter assay was performed to verify the binding relationship between miR-185-5p and YWHAZ in NCI-H1299 and PC9 cells. **C** and **D** The mRNA level of YWHAZ was measured in NCI-H1299 and PC9 cells by qRT-PCR after miR-185-5p mimic (**C**) or inhibitor (**D**) transfection. **E** Western blot was performed to measure the protein level of YWHAZ in NCI-H1299 and PC9 cells transfected with miR-185-5p mimic. **F** The mRNA expression level of YWHAZ in NSCLC tissues. **G** The correlation analysis between YWHAZ and miR-185-5p expression level. Each experiments included three biological replicates. The data was displayed as Mean ± SD. **p < 0.01, ***p < 0.001

We employed bioinformatics tools to anticipate probable miR-185-5p downstream targets. According to Starbase2.0 data, YWHAZ 3’-UTR has one potential binding site to miR-185-5p. Dual-luciferase reporter assay was performed to study the potential interaction between YWHAZ 3′-UTR with miR-185-5p. After co-transfecting YWHAZ-WT or YWHAZ-MUT with miR-185-5p mimic or miR-NC into NCI-H1299 and PC9 cells, miR-185-5p significantly decreased the relative luciferase activity of YWHAZ-WT but not YWHAZ-MUT transfected cells (*p* < 0.01, Fig. 3B). The data before showed the binding relationship between miR-185-5p and YWHAZ 3′-UTR.

To further verify the binding relationship, we detected the mRNA and protein levels of YWHAZ in the NSCLC cell lines transfected with miR-185-5p mimic, miR-185-5p inhibitor or miR-NC respectively. As observed in Fig. 3C and D, overexpression of miR-185-5p obviously reduced the mRNA level of YWHAZ (*p* < 0.001), while the knockdown of miR-185-5p reversed the effect of miR-185-5p on YWHAZ expression. Consistently, overexpression of miR-185-5p substantially decreased the protein level of YWHAZ both in NCI-H1299 and PC9 cells (*p* < 0.001, Fig. 3E). Moreover, YWHAZ was significantly up-regulated in NSCLC tumors (Fig. 3F). The results of Spearman's correlation analysis showed a negative correlation between the expression of YWHAZ and miR-185-5p in NSCLC tumors (Fig. 3G). Collectively, these findings revealed that YWHAZ was a direct downstream target of miR-185-5p and was regulated by miR-185-5p negatively in NSCLC.

### YWHAZ overexpression reverses the effects of miR-185-5p overexpression on NSCLC cells

**Fig. 4:**
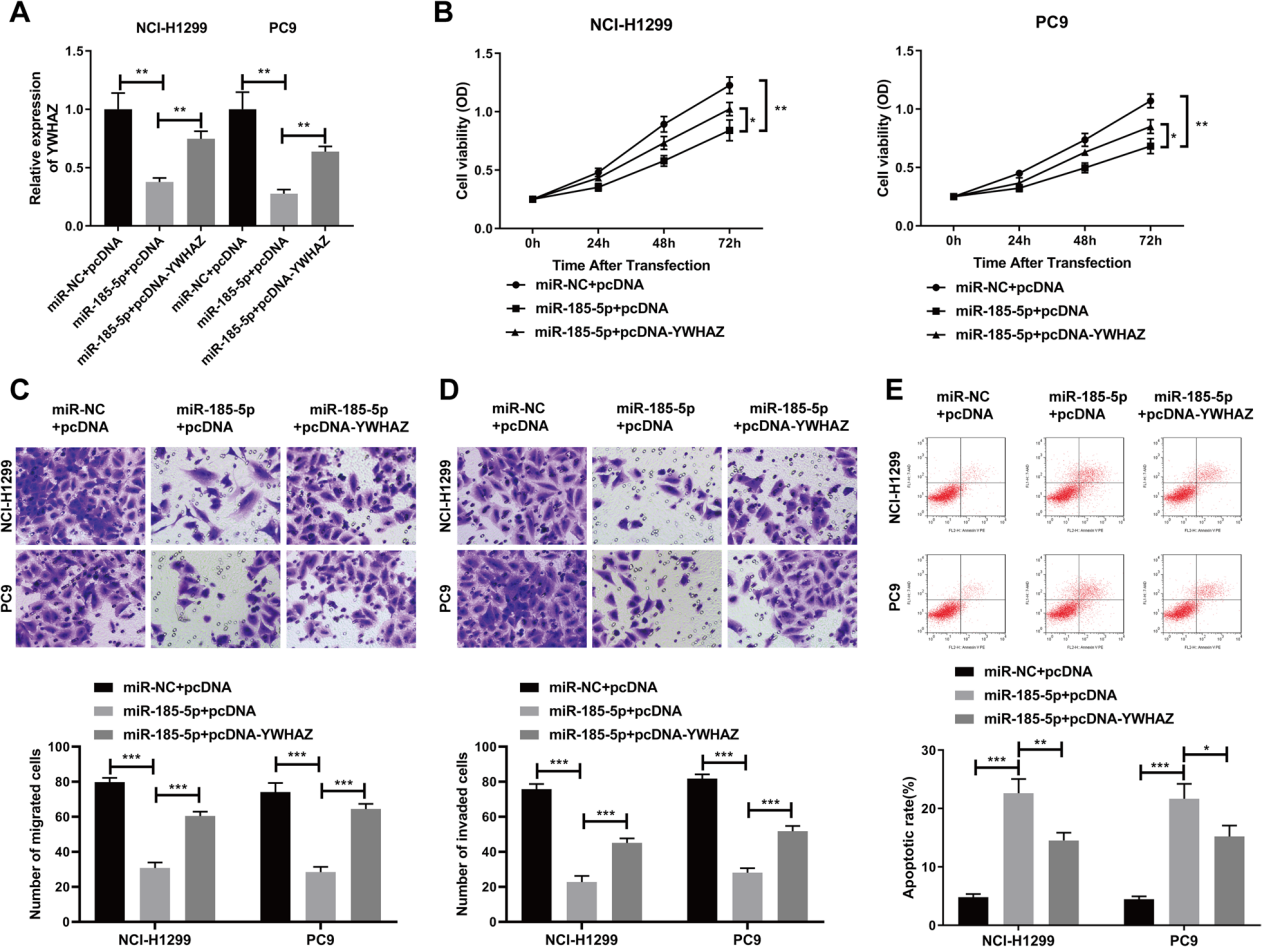
YWHAZ overexpression reverses the effects of miR-185-5p overexpression on NSCLC cells. **A** QRT-PCR was performed to assess the transfection efficiency. **B** CCK-8 assay was used to evaluate the proliferation ability of NCI-H1299 and PC9 after different treatment. **C** and **D** Transwell assay was used to assess the migration (**C**) and invasion (**D**) ability of NCI-H1299 and PC9 after different treatment. **E** Cell apoptosis was measured by flow cytometry in NCI-H1299 and PC9 after different treatment. Each experiments included three biological replicates. The data was displayed as Mean ± SD. *p < 0.05, **p < 0.01, ***p < 0.001

Since miR-185-5p overexpression suppressed the cell proliferation, migration and invasion, promoted cell apoptosis of NSCLC cells and inhibited YWHAZ expression, we predicted that miR-185-5p mediated inhibitory effect on NSCLC cells was related to YWHAZ. Thus, we constructed the YWHAZ overexpression vector (pcDNA-YWHAZ) to up-regulate its expression. Firstly, NCI-H1299 and PC9 cells were co-transfected with pcDNA-YWHAZ vector and miR-185-5p. The result of RT-qPCR assay showed that the deleption of YWHAZ mediated by miR-185-5p transfection was obviously rescued by pcDNA-YWHAZ vector transfection in NCI-H1299 and PC9 cells (Fig. 4A). Then, we performed the CCK-8, transwell and flow cytometry assay, respectively, to see if miR-185-5p influenced NSCLC cell proliferation, invasion, migration, and apoptosis by regulating YWHAZ expression. As shown in Fig. 4B, overexpression of miR-185-5p suppressed the ability of cell proliferation (*p* < 0.01), but overexpression of YWHAZ significantly rescued the inhibitory effect of miR-185-5p on proliferation of H1299 and PC9 cells (*p* < 0.05). Furthermore, we found that the migration and invasion capabilities were inhibited in miR-185-5p alone-transfected NSCLC cells and this phenomenon was partially reversed by co-transfecting with pcDNA-YWHAZ vector and miR-185-5p (Fig. 4C, D). Consistently, the flow cytometry assay indicated that the co-transfection with miR-185-5p and pcDNA-YWHAZ inhibited the promotion of cell apoptosis caused by the overexpression of miR-185-5p in NSCLC cells (Fig. 4E). All in all, these results suggested that YWHAZ overexpression reversed the effects of miR-185-5p overexpression on NSCLC cells.

### The effect of miR-185-5p on tumor growth in mice

**Fig. 5:**
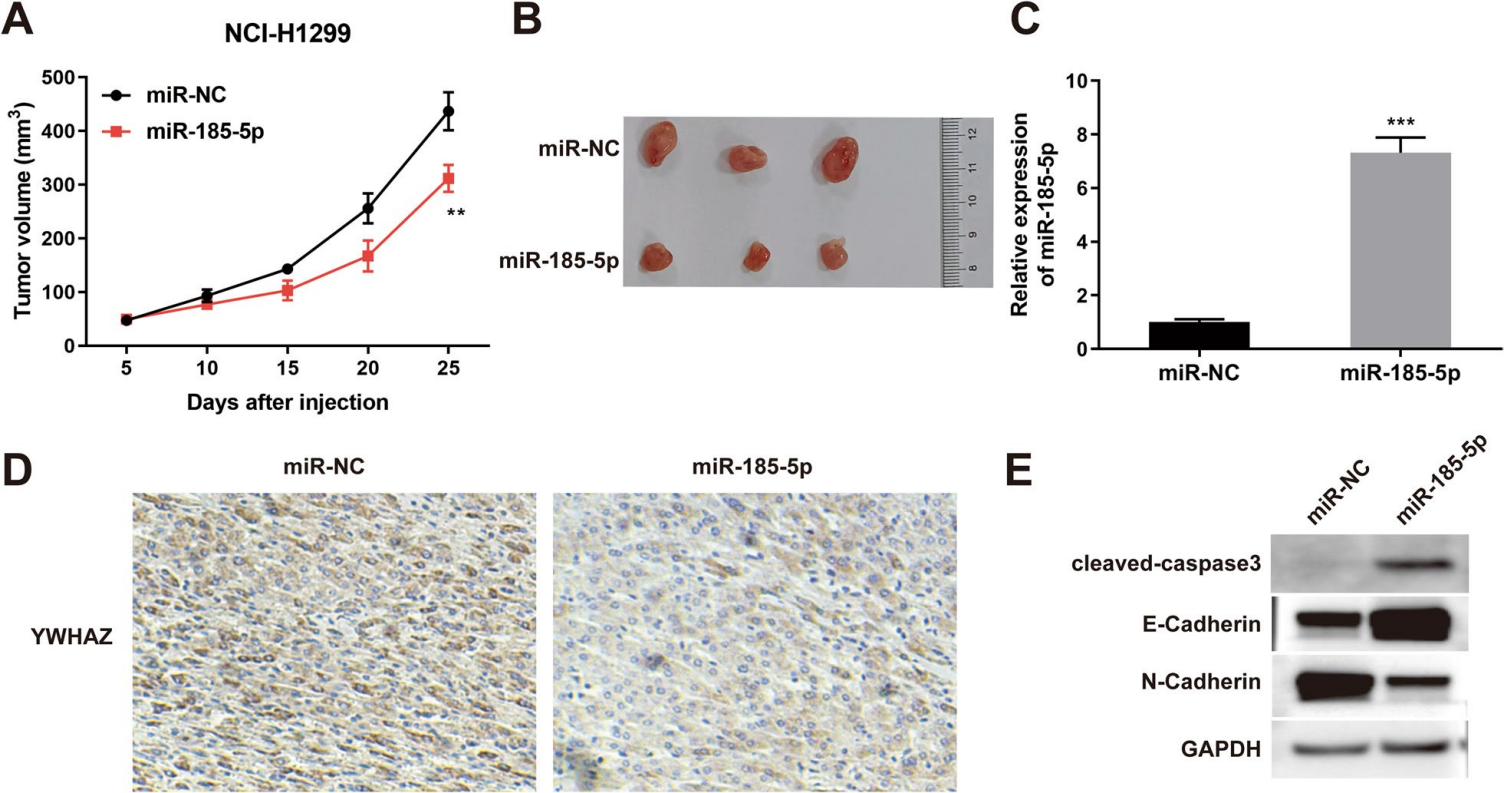
The effect of miR-185-5p on tumor growth in mice. **A** The tumor volume was measured every 5 days before sacrificed. **B** The image of tumors. **C** QRT-PCR was performed to evaluate miR-185-5p expression in tumors. **D** YWHAZ expression was detected by Immunohistochemistry assay. **E** E-Cadherin, N-Cadherin and cleaved-caspase3 expression were measured by western blot in tumor tissues. Each experiments included three biological replicates. The data was displayed as Mean ± SD. **P<0.01, ***P<0.001

Given that miR-185-5p overexpression was confirmed that inhibited cell biological behaviors in NSCLC cells in vitro, we assessed whether the up-regulation of miR-185-5p might affect tumor growth in vivo subsequently. First, we subcutaneously injected NCI-H1299 cells stably transfected with miR-185-5p mimic or miR-NC into the left flanks of mice. The tumors from mice of each group (miR-185-5p group and miR-NC group) were excised after the mice were sacrificed. As shown in Fig. 5A and B, overexpression of miR-185-5p in miR-185-5p group led to a marked reduction of tumor volume compared with miR-NC group (*p* < 0.05). Then, the expression level of miR-185-5p was examined in tumor tissues by qRT-PCR. In comparison to the miR-NC group, the miR-185-5p group showed a significant increase in miR-185-5p expression (*p* < 0.001, Fig. 5C). Moreover, we performed an immunohistochemistry assay to assess the YWHAZ expression in tumor tissues. The expression of YWHAZ was lower in tumor tissues of the miR-185-5p group than in the miR-NC group (Fig. 5D). The results showed that miR-185-5p inhibited YWHAZ expression in tumor tissues, which was in line with the results of previous cell tests. Besides, western blot analysis indicated that overexpression of miR-185-5p, increased the expression of cleaved-Caspase3, but induced E-cadherin up-regulation and N-cadherin down-regulation in tumor tissues (Fig. 5E). Taken together, miR-185-5p inhibited tumor growth in mice.

## Discussion

Lung cancer is one of the most serious diseases that endanger human health around the world. NSCLC is the predominant type of lung cancer, accounting for approximately 85% of lung cancer-related deaths. Despite recent advances in surgical treatment, chemotherapy, radiation, and molecular targeted therapy for NSCLC, the 5-year overall survival rate for individuals with NSCLC is still less than 15% [14]. According to numerous studies, miRNAs play a crucial role in a series of biological processes including tumorigenesis. Identifying miRNAs implicated in the NSCLC progression will help to understand its mechanism and help the treatment of NSCLC [15]. For example, miR-187 overexpression inhibited growth and colony formation ability of NSCLC cells by binding directly to FGF9 [16]. In addition, upregulation of miR-25 was correlated with lymph node metastasis and TNM stage in NSCLC. The restoration of CDH1 expression-inhibited cell invasion and migration induced by miR-25 overexpression [17]. These findings suggest that miRNAs are potential molecular markers and therapeutic targets for NSCLC.

Notably, studies have demonstrated that miR-185 has a tumor suppressor function and induces G1 cell cycle arrest in NSCLC [18, 19]. At the same time, Low expression of miR-185-5p in NSCLC has been documented, which is an individual risk factor for poor patient prognosis [10]. Bian et al. reported that down-regulated circCDYL promoted cell apoptosis through the miR-185-5p/TNRC6A axis in NSCLC [20]. In this study, we verified the low expression of miR-185-5p and the effect on biological behaviors of cells (enhanced cell proliferation, migration, invasion, and weakened cell apoptosis) in NSCLC. In addition, we predicted and confirmed that YWHAZ was the target gene of miR-185-5p by bioinformatics analysis software StarBase2.0 and dual luciferase reporter gene experiments, and YWHAZ overexpression plasmid reversed the regulatory effects of miR-185-5p on NSCLC cells.

YWHAZ is a molecular family of proteins with a molecular weight of approximately 30 kDa and a highly conserved structure [21, 22]. As a central protein involved in many signal transduction pathways, YWHAZ plays a key role in tumor progression. More and more studies have shown that YWHAZ is upregulated in various types of cancer, such as hepatocellular carcinoma, colorectal cancer, lung cancer and breast cancer, and act as an oncogene to promote the malignant behavior of tumor cells, including cell growth, cell cycle, apoptosis, migration/invasion [23–26]. Furthermore, YWHAZ has been reported to be regulated by miRNAs or long noncoding RNAs (lncRNAs) to exert its malignant functions, which may serve as potential biomarkers for the diagnosis, prognosis and chemoresistance of several cancers. The combination of YWHAZ with other cancer-specific molecules may have better capacity as a biomarker. However, only a few studies have reported the underlying mechanism of YWHAZ in NSCLC progression. For example, Jia et al. reported that the important regulator MLK7-AS1 in NSCLC promoted cell invasion in vitro and in vivo by upregulating the miR-375-3p/YWHAZ axis [27]. Zhao et al. found that 14-3-3ζ and Hsp27 worked together to promote NSCLC progression by participating in TGF-β-induced tumor development [28]. At present, the molecular mechanism of YWHAZ in the progression of NSCLC remains unclear.

In this study, YWHAZ was confirmed as a target gene of miR-185-5p. Overexpression of miR-185-5p suppressed YWHAZ expression, while the knockdown of miR-185-5p reversed the effect of miR-185-5p on YWHAZ expression in NSCLC. The results of experiments in vitro showed that the effect of miR-185-5p overexpression on the biological behaviors of NSCLC cells, including weakened cell proliferation, invasion migration and enhanced cell apoptosis, could be partially reversed by transfecting YWHAZ overexpression vector into NSCLC cells. Meanwhile, we constructed the xenograft tumor model by injecting NCI-H1299 cells stably transfected with miR-185-5p into nude mice for in vivo experiments. The in vivo results were consistent with the in vitro results, which showed that miR-185-5p overexpression negatively regulated the expression of YWHAZ and inhibited tumor growth. Therefore, these results suggested that miR-185-5p regulated the progression of NSCLC by regulating YWHAZ expression.

In conclusion, our results showed that miR-185-5p was down-regulated in tumor tissues and cell lines of NSCLC, and that miR-185-5p overexpression inhibited NSCLC cell proliferation, migration and invasion, and enhanced cell proliferation apoptosis, and suppressed tumor growth by negatively regulating YWHAZ. Therefore, these findings may provide the basis for innovative treatment strategies for NSCLC.

## Conclusion

In summary, miR-185-5p was significantly down-regulated in NSCLC, and that the overexpression of miR-185-5p inhibited malignant behaviors of cells and tumor growth by negatively regulating YWHAZ. Therefore, the results of this research suggested that miR-185-5p could be a viable therapeutic target for NSCLC and revealed a novel mechanism in NSCLC progression.

## Data Availability

The data used to support the findings of this study are available from the corresponding author upon request.
